# An Efficient Computational Technique for Fractal Vehicular Traffic Flow

**DOI:** 10.3390/e20040259

**Published:** 2018-04-09

**Authors:** Devendra Kumar, Fairouz Tchier, Jagdev Singh, Dumitru Baleanu

**Affiliations:** 1 Department of Mathematics, JECRC University, Jaipur 303905, India; 2 Department of Mathematics, King Saud University, P.O. Box 22452, Riyadh 11495, Saudi Arabia; 3 Department of Mathematics, Cankaya University, Balgat, Ankara 06530, Turkey; 4 Institute of Space Sciences, Magurele-Bucharest 077125, Romania

**Keywords:** fractal vehicular traffic flow, local fractional Sumudu transform, homotopy perturbation technique, reduced differential transform method, local fractional derivative

## Abstract

In this work, we examine a fractal vehicular traffic flow problem. The partial differential equations describing a fractal vehicular traffic flow are solved with the aid of the local fractional homotopy perturbation Sumudu transform scheme and the local fractional reduced differential transform method. Some illustrative examples are taken to describe the success of the suggested techniques. The results derived with the aid of the suggested schemes reveal that the present schemes are very efficient for obtaining the non-differentiable solution to fractal vehicular traffic flow problem.

## 1. Introduction

Firstly, the continuum model was used to elucidate a traffic flow with continuous functions, which was similar to one of fluid dynamics and material models established based on conservation laws. Connected to this, pioneering work was conducted by Lighthill and Whitham [1] and Richards [2] who formulated a model named the Lighthill-Whitham-Richards’s (LWR) model. This mathematical model was examined by many research workers [3,4,5,6].

Similar to integer order models, fractional order models are very useful, with extra advantages because they consider full memory effect. Consequently, many researchers have applied this innovative approach of fractional calculus in the mathematical modeling of natural phenomena. Among these are Machado and Mata [7], Carvalho and Pinto [8], Zhou et al. [9], Kumar et al. [10,11], Singh et al. [12], Hristov [13], Yang et al. [14], Area et al. [15], Zaky and Machado [16], Drapaca and Sivaloganathan [17], Sumelka et al. [18], Lazopoulos and Lazopoulos [19], Rahimi et al. [20], and others. In recent years an innovative and very interesting theory of fractional calculus, namely local fractional calculus, has earned importance and popularity among scientists working in this special branch [21,22]. Today, local fractional derivatives and integrals has been utilized to describe various scientific and technological problems, such as local fractional 2-D Burgers-type equations [23], nonlinear Riccati differential equations containing local fractional derivatives [24], diffusion and heat equations pertaining to local fractional operators [25], local fractional Burgers equations [26] and many other problems. In view of the great usefulness of local fractional calculus, we examine the local fractional LWR model on a finite-length highway arising in vehicular traffic flow written as

(1) $$\frac{\partial^{\beta }w(r,s)}{\partial s^{\beta }}+\lambda \frac{\partial^{\beta }w(r,s)}{\partial r^{\beta }}=0,0<\beta \leq 1,$$

surrounding the initial and boundary conditions

(2) $$w(r,0)=g_{1}(r),$$

(3) $$w(0,s)=g_{2}(s).$$

The local fractional LWR model with finite-length highway has been examined by the local fractional Laplace variational iteration scheme [27], the local fractional Laplace decomposition approach and the local fractional series expansion scheme [28]. In the present work, we examine the local fractional LWR model by the local fractional homotopy perturbation Sumudu transform method (LFHPSTM) [29,30,31]. Furthermore, we analyze the local fractional LWR model with the aid of local fractional reduced differential transform method (LFRDTM) [32,33]. The LFHPSTM is a hybrid scheme and is developed by the mixing of LFHPM [34,35,36] and a local fractional Sumudu transform technique [37].

The rest of this article is presented as follows: Section 2 involves the primary properties of local fractional calculus. Section 3 is devoted to the solution procedure of LFHPSTM. In Section 4, the solution procedure of LFRDTM is discussed. Section 5 is dedicated to the solution of the local fractional LWR model with a finite-length highway. At the end, Section 6 is devoted to concluding remarks.

## 2. Local Fractional Calculus and Its Properties

Here, we highlight the main concept of local fractional calculus, which is employed in the present research work.

**Definition** **1.** [21,22,23,24,25,26,27,28,29]. *Let us assume a function* $w(s)\in C_{\beta }(\tau ,\upsilon ),$ *while*

(4) $$|w(s)-w(s_{0})|<\varepsilon^{\beta },\;0<\beta \leq 1,$$

*is valid, when* $\left|s-s_{0}\right|<\delta ,$ *for* ε, δ>0 *and* ε∈ℜ.

**Definition** **2.** [21,22,23,24,25,26,27,28,29]. *Let us assume the interval* [τ,υ] *and* $(s_{j},s_{j+1}),$ j=0,…,N−1, $s_{0}=\tau ,$ *and* $s_{N}=\upsilon$ *with* $\Delta s_{j}=s_{j+1}-s_{j}$, $\Delta s=\max\{\Delta s_{0},\Delta s_{1,},\Delta s_{2,},\ldots \}$ *be partition of* [τ,υ]. *Then local fractional integral operator of* $w(s)\in C_{\beta }(\tau ,\upsilon )$ *is expressed in the subsequent manner*

(5) Iυτ(β)w(s)=1Γ(1+β)∫τυw(s)(ds)β=1Γ(1+β)LimΔs→0w(Δsj)β.

**Definition** **3.** [21,22,23,24,25,26,27,28,29]. *Let* w(s) *hold the condition presented in Equation (4), then the inverse formula for Equation (5) is presented in the subsequent manner:*

(6) $$\frac{d^{\beta }w(s_{0})}{ds^{\beta }}=D_{s}^{\left(\beta \right)}w(s_{0})=\frac{\Delta^{\beta }\left(w(s)-w(s_{0})\right)}{{\left(s-s_{0}\right)}^{\beta }},$$

*where*

(7) $$\Delta^{\beta }\left(w(s)-w(s_{0})\right)≅\Gamma (1+\beta )[w(s)-w(s_{0})].$$

**Definition** **4.** *The local fractional Sumudu transform (LFST) [37] is an extension of Sumudu transform [38] and LFST of a function* w(s) *is expressed as*

(8) $$\begin{array}{l} LFS_{\beta }\left\{w(s)\right\}=W_{\beta }(z)\\ =\frac{1}{\Gamma (1+\beta )}\int_{0}^{\infty }E_{\beta }(-z^{-\beta }s^{\beta })\frac{w(s)}{z^{\beta }}{\left(ds\right)}^{\beta },\beta \in (0,1]. \end{array}$$

*The inverse formula for LFST is defined as*

(9) $$LFS_{\beta }^{-1}\left\{W(z)\right\}=w(s),0<\beta \leq 1.$$

Further important results and useful properties of this transform can be seen in the work of Belgacem et al. [39].

**Definition** **5.** *The Mittag-Leffler function* [40] *is presented as*

(10) Eβ(s)=∑m=0∞smΓ(βm+1), (β∈C, Re(β)>0).

## 3. Basic Idea LFHPSTM

Here we discuss the main outline of the LFHPSTM. Let us take a linear differential equation associated with the local fractional derivative of the form

(11) $$L_{\beta }w(r,s)+R_{\beta }w(r,s)=q(r,s).$$

In Equation (11) $L_{\beta }$ represents the linear local fractional differential operator, $R_{\beta }$ indicates the rest part of the linear operator and q(r,s) represents a function known as source function.

By using the LFST on Equation (11), it yields

(12) $$W(r,z)=w(r,0)+z^{\beta }w^{\left(\beta \right)}(r,0)+z^{2\beta }w^{\left(2\beta \right)}(r,0)+\ldots \;+z^{(k-1)\beta }w^{\left((k-1)\beta \right)}(r,0)-z^{k\beta }LFS_{\beta }\left[R_{\beta }w(r,s)\right]+z^{k\beta }LFS_{\beta }\left[q(r,s)\right].$$

Applying the inverse of LFST on Equation (12), we get

(13) $$w(r,s)=S(r,s)-LFS_{\beta }^{-1}{\left[z^{k\beta }LFS_{\beta }\left[R_{\beta }w(r,s)\right]\right]}_{}^{}.$$

In Equation (13) the term S(r,s) occurs due to initial conditions and source function.

Now the LFHPM [36] is employed as

(14) $$w(r,s)=\sum_{m=0}^{\infty }p^{m\beta }w_{m}(r,s).$$

By making use of Equation (14) in Equation (13), we get

(15) $$\sum_{m=0}^{\infty }p^{m\beta }w_{\beta ,m}(r,s)=S(r,s)-p^{\beta }LFS_{\beta }^{-1}\left[z^{k\beta }LFS_{\beta }\left[R_{\beta }\sum_{m=0}^{\infty }p^{m\beta }w_{m}(r,s)\right]\right].$$

The result (15) is derived by a combination the LFST and LFHPM.

The comparison of the coefficients of the equal powers of *p* in both sides gives

$$p^{0\beta }:w_{0}(r,s)=S(r,s)$$

$$p^{1\beta }:w_{1}(r,s)=-LFS_{\beta }^{-1}\left[z^{k\beta }LFS_{\beta }\left[R_{\beta }w_{0}(r,s)\right]\right],$$

$$p^{2\beta }:w_{2}(r,s)=-LFS_{\beta }^{-1}\left[z^{k\beta }LFS_{\beta }\left[R_{\beta }w_{1}(r,s)\right]\right],$$

Thus, the LFHPSTM solution is expressed as follows

(16) $$w(r,s)=\underset{M\to \infty }{\lim}\sum_{m=0}^{M}w_{m}(r,s).$$

## 4. Basic Idea of LFRDTM

Here, we present the basic definitions and properties of LFRDTM [32,33].

**Lemma** **1 (Local fractional Taylors theorem).** *[32,33]. Let us consider that* $\frac{d^{(k+1)\beta }}{dr^{(k+1)\beta }}w(r)\in C_{\beta }(\tau ,\upsilon ),$ *for* τ,υ∈ℜ,k=0,1,2,…,n *and* 0<β≤1, *we have*

(17) $$w(r)=\sum_{k=0}^{\infty }\frac{d^{k\beta }}{dr^{k\beta }}w(r_{0})\frac{{\left(r-r_{0}\right)}^{k\beta }}{\Gamma (1+k\beta )}.$$

*In Equation (17)* $\tau <r_{0}<r<\upsilon ,\forall r\in (\tau ,\upsilon ).$

**Definition** **6.** *The LFRDT* $W_{k}(r)$ *of a function* w(r,s) *is expressed in the following manner [32,33]*

(18) $$W_{k}(r)=\frac{1}{\Gamma (1+k\beta )}{\left[\frac{\partial^{k\beta }}{\partial s^{k\beta }}w(r,s)\right]}_{s=s_{0}}.$$

*In Equation (28)* k=0,1,2,…,n *and* 0<β≤1.

**Definition** **7.** *The LFRDT of* $W_{k}(r)$ *is expressed in the following manner [32,33]*

(19) $$w(r,s)=\sum_{k=0}^{\infty }W_{k}(r){\left(s-s_{0}\right)}^{k\beta },0<\beta \leq 1.$$

The fundamental mathematical operations of the LFRDTM [32,33] are presented in Table 1.

## 5. Non-Differential Solutions for Local Fractional LWR Model on a Finite Length Highway

Here, we derive the non-differential solutions of the local fractional LWR model by using LFHPSTM and LFRDTM.

**Example** **1.** *Firstly, we examine the following local fractional LWR model having finite-length highway*

(20) $$\frac{\partial^{\beta }w(r,s)}{\partial s^{\beta }}+\lambda \frac{\partial^{\beta }w(r,s)}{\partial r^{\beta }}=0,0<\beta \leq 1,$$

*surrounding the initial and boundary conditions*

(21) $$w(r,0)=E_{\beta }(r^{\beta })$$

(22) $$w(0,s)=\cosh_{\beta }(\lambda s^{\beta })-\sinh_{\beta }(\lambda s^{\beta })$$

*On applying the LFST on Equation (20) and simplifying, we have*

(23) $$W(r,z)=E_{\beta }(r^{\beta })-\lambda z^{\beta }LFS_{\beta }\left[\frac{\partial^{\beta }w(r,s)}{\partial r^{\beta }}\right].$$

*On employing the inverse of LFST on Equation (23), we get*

(24) $$w(r,s)=E_{\beta }(r^{\beta })-\lambda LFS_{\beta }^{-1}\left[z^{\beta }LFS_{\beta }\left[\frac{\partial^{\beta }w(r,s)}{\partial r^{\beta }}\right]\right].$$

*Now by employing LFHPM [36], we have*

(25) $$\sum_{m=0}^{\infty }p^{m\beta }w_{m}(r,s)=E_{\beta }(r^{\beta })-\lambda p^{\beta }LFS_{\beta }^{-1}\left[z^{\beta }LFS_{\beta }\left[\frac{\partial^{\beta }\left(\sum_{m=0}^{\infty }p^{m\beta }w_{m}(r,s)\right)}{\partial r^{\beta }}\right]\right].$$

*On comparing the like powers of p, it yields*

(26) $$p^{0\beta }:w_{0}(r,s)=E_{\beta }(r^{\beta }),\;p^{1\beta }:w_{1}(r,s)=-\frac{\lambda s^{\beta }}{\Gamma (1+\beta )}E_{\beta }(r^{\beta }),\;p^{2\beta }:w_{2}(r,s)=\frac{\lambda^{2}s^{2\beta }}{\Gamma (1+2\beta )}E_{\beta }(r^{\beta }).$$

*By making use of the same operations, we evaluate the rest of terms of the LFHPSTM solution. On that account, the solution of local fractional LWR model (20) is expressed in the subsequent manner*

(27) $$w(r,s)=\sum_{m=0}^{\infty }w_{m}(r,s)\;=E_{\beta }(r^{\beta })\left[1+\frac{\lambda^{2}s^{2\beta }}{\Gamma (1+2\beta )}+\frac{\lambda^{4}s^{4\beta }}{\Gamma (1+4\beta )}+\cdots \right]-E_{\beta }(r^{\beta })\left[\frac{\lambda s^{\beta }}{\Gamma (1+\beta )}+\frac{\lambda^{3}s^{3\beta }}{\Gamma (1+3\beta )}+\cdots \right].$$

*It can be indicated in closed form in the subsequent manner*

(28) $$w(r,s)=E_{\beta }(r^{\beta })\cosh_{\beta }(\lambda s^{\beta })-E_{\beta }(r^{\beta })\sinh_{\beta }(\lambda s^{\beta }).$$

Now, we apply the LFRDTM on local fractional LWR model.

*On applying the LFRDT on Equation (20), it gives*

(29) $$\frac{\Gamma (k\beta +\beta +1)}{\Gamma (k\beta +1)}W_{k+1}(r)=-\lambda \frac{\partial^{\beta }W_{k}(r)}{\partial r^{\beta }}.$$

*In Equation (29)* $W_{k}(r)$ *represents the transformed function. From the initial condition (21), we can express as*

(30) $$W_{0}(r)=E_{\beta }(r^{\beta }).$$

*On using Equation (30) in Equation (29) and iterative steps, we get*

(31) $$\begin{array}{c} W_{1}(r)=-E_{\beta }(r^{\beta })\frac{\lambda }{\Gamma (1+\beta )},W_{2}(r)=E_{\beta }(r^{\beta })\frac{\lambda^{2}}{\Gamma (1+2\beta )},\ldots \\ W_{n}(r)={\left(-1\right)}^{n}E_{\beta }(r^{\beta })\frac{\lambda^{n}}{\Gamma (1+n\beta )},\ldots \end{array}$$

*Therefore, LFRDTM solution is given by*

$$w(r,s)=\sum_{k=0}^{\infty }W_{k}(r)s^{k\beta }$$

(32) $$=E_{\beta }(r^{\beta })\left[1+\frac{\lambda^{2}s^{2\beta }}{\Gamma (1+2\beta )}+\frac{\lambda^{4}s^{4\beta }}{\Gamma (1+4\beta )}+\cdots \right]-E_{\beta }(r^{\beta })\left[\frac{\lambda s^{\beta }}{\Gamma (1+\beta )}+\frac{\lambda^{3}s^{3\beta }}{\Gamma (1+3\beta )}+\cdots \right].$$

*It can be expressed in closed form in the subsequent manner*

(33) $$w(r,s)=E_{\beta }(r^{\beta })\cosh_{\beta }(\lambda s^{\beta })-E_{\beta }(r^{\beta })\sinh_{\beta }(\lambda s^{\beta }).$$

From the results (28) and (33), we can see the results obtained by using LFHPSTM and LFRDTM are in good agreement and give the exact solution in term of Mittag-Leffler function, which is suitable for numerical computation.

**Example** **2.** *Next, we examine the following local fractional LWR model having finite length highway*

(34) $$\frac{\partial^{\beta }w(r,s)}{\partial s^{\beta }}+\frac{\partial^{\beta }w(r,s)}{\partial r^{\beta }}=0,0<\beta \leq 1,$$

*surrounding the initial and boundary conditions*

(35) $$w(r,0)=\sinh_{\beta }(r^{\beta }),$$

(36) $$w(0,s)=-\sinh_{\beta }(s^{\beta }).$$

*On employing the LFST on Equation (34) and simplifying, we arrive at the subsequent result*

(37) $$W(r,z)=\sinh_{\beta }(r^{\beta })-z^{\beta }LFS_{\beta }\left[\frac{\partial^{\beta }w(r,s)}{\partial r^{\beta }}\right].$$

*On employing the inverse of LFST on Equation (37), we get*

(38) $$w(r,s)=\sinh_{\beta }(r^{\beta })-LFS_{\beta }^{-1}\left[z^{\beta }LFS_{\beta }\left[\frac{\partial^{\beta }w(r,s)}{\partial r^{\beta }}\right]\right].$$

*Next, we employ the LFHPM [36], it gives*

(39) $$\sum_{m=0}^{\infty }p^{m\beta }w_{m}(r,s)=\sinh_{\beta }(r^{\beta })-p^{\beta }LFS_{\beta }^{-1}\left[z^{\beta }LFS_{\beta }\left[\frac{\partial^{\beta }\left(\sum_{m=0}^{\infty }p^{m\beta }w_{m}(r,s)\right)}{\partial r^{\beta }}\right]\right].$$

*Comparing the same powers of p, we have*

(40) $$p^{0\beta }:w_{0}(r,s)=\sinh_{\beta }(r^{\beta }),\;p^{1\beta }:w_{1}(r,s)=-\frac{s^{\beta }}{\Gamma (1+\beta )}\cosh_{\beta }(r^{\beta }),\;p^{2\beta }:w_{2}(r,s)=\frac{s^{2\beta }}{\Gamma (1+2\beta )}\sinh_{\beta }(r^{\beta }).$$

*By making use of the same operations, we compute the rest of terms of the LFHPSTM solution. Consequently, the solution of local fractional LWR model (34) is expressed as*

(41) $$w(r,s)=\sum_{m=0}^{\infty }w_{m}(r,s)\;=\sinh_{\beta }(r^{\beta })\left[1+\frac{s^{2\beta }}{\Gamma (1+2\beta )}+\frac{s^{4\beta }}{\Gamma (1+4\beta )}+\cdots \right]-\cosh_{\beta }(r^{\beta })\left[\frac{s^{\beta }}{\Gamma (1+\beta )}+\frac{s^{3\beta }}{\Gamma (1+3\beta )}+\cdots \right].$$

*It can be represented in closed form in the subsequent manner*

(42) $$w(r,s)=\sinh_{\beta }(r^{\beta })\cosh_{\beta }(s^{\beta })-\cosh_{\beta }(r^{\beta })\sinh_{\beta }(s^{\beta }).$$

Next, we use the LFRDTM to study the local fractional LWR model (34).

*On applying the LFRDT on Equation (34), it yields*

(43) $$\frac{\Gamma (k\beta +\beta +1)}{\Gamma (k\beta +1)}W_{k+1}(r)=-\frac{\partial^{\beta }W_{k}(r)}{\partial r^{\beta }}.$$

*In Equation (43)* $W_{k}(r)$ *represents the transformed function. From the initial condition (35), we can express as*

(44) $$W_{0}(r)=\sinh_{\beta }(r^{\beta }).$$

*On using Equation (44) in Equation (43) and iterative steps, we have*

(45) $$\begin{array}{c} W_{1}(r)=-\cosh_{\beta }(r^{\beta })\frac{1}{\Gamma (1+\beta )},W_{2}(r)=\sinh_{\beta }(r^{\beta })\frac{1}{\Gamma (1+2\beta )},\\ W_{3}(r)=-\cosh_{\beta }(r^{\beta })\frac{1}{\Gamma (1+3\beta )}\ldots \end{array}$$

*Therefore, LFRDTM solution is presented as*

(46) $$w(r,s)=\sum_{k=0}^{\infty }W_{k}(r)s^{k\beta }\;=\sinh_{\beta }(r^{\beta })\left[1+\frac{s^{2\beta }}{\Gamma (1+2\beta )}+\frac{s^{4\beta }}{\Gamma (1+4\beta )}+\cdots \right]-\cosh_{\beta }(r^{\beta })\left[\frac{s^{\beta }}{\Gamma (1+\beta )}+\frac{s^{3\beta }}{\Gamma (1+3\beta )}+\cdots \right].$$

*It can be represented in closed form in the subsequent manner*

(47) $$w(r,s)=\sinh_{\beta }(r^{\beta })\cosh_{\beta }(s^{\beta })-\cosh_{\beta }(r^{\beta })\sinh_{\beta }(s^{\beta }).$$

From the results (42) and (47), we can see the results obtained by using LFHPSTM and LFRDTM are in good agreement and give the exact solution in closed form which is suitable for numerical computation.

## 6. Concluding Remarks

In this research article, we have analyzed the local fractional LWR model on a finite-length highway. The LFHMSTM and LFRDTM are used to obtain the non-differentiable solution of local fractional LWR model and the corresponding solutions are presented in closed form, which are very suitable for numerical computation. The result indicates that the suggested computational schemes are very simple and computationally sound for handling similar kinds of differential equations occurring in natural sciences.

## Figures and Tables

**Table 1:** Reduced differential transformations.

Original Function	LFRDT Function
w(r,s)	$W_{k}(r)=\frac{1}{\Gamma (k\beta +1)}{\left[\frac{\partial^{k\beta }}{\partial s^{k\beta }}w(r,s)\right]}_{s=0}$
$w(r,s)=c_{1}\psi (r,s)\pm c_{2}\varphi (r,s)$	$W_{k}(r)=c_{1}\Psi_{k}(r)\pm c_{2}\Phi_{k}(r)$
w(r,s)=ψ(r,s)φ(r,s)	$W_{k}(r)=\sum_{i=0}^{k}\Psi_{i}(r)\Phi_{k-i}(r)$
$w(r,s)=(\partial^{n\beta }/\partial s^{n\beta })\psi (r,s)$	$W_{k}(r)=[(\Gamma (k\beta +n\beta +1))/\Gamma (k\beta +1))]\Psi_{k+n}(r)$
$w(r,s)=\frac{{\left(r-r_{0}\right)}^{m\beta }}{\Gamma (1+m\beta )}\frac{{\left(s-s_{0}\right)}^{n\beta }}{\Gamma (1+n\beta )}$	$W_{k}(r)=\frac{r^{m\beta }}{\Gamma (1+m\beta )}\delta_{\beta }(k-n)$
$w(r,s)=E_{\beta }({\left(a(r-r_{0})\right)}^{\beta })E_{\alpha }({\left(b(s-s_{0})\right)}^{\alpha })$	$W_{k}(r)=E_{\beta }({\left(a(r-r_{0})\right)}^{\beta })\frac{a^{k\beta }}{\Gamma (1+k\beta )}$

## References

[1] Lighthill M.J., Whitham G.B. (1955). On kinematic waves-II. A theory of traffic flow on long crowded roads. Proc. Roy. Soc. London Ser. A.

[2] Richards P.I. (1956). Shock waves on the highway. Oper. Res..

[3] Daganzo C.F. (1997). A continuum theory of traffic dynamics for freeways with special lanes. Transp. Res. Part B Methodol..

[4] Bellomo N., Delitala M., Coscia V. (2002). On the mathematical theory of vehicular traffic flow—I: Fluid dynamic and kinetic modelling. Math. Models Methods Appl. Sci..

[5] Zhang H.M. (2001). New perspectives on continuum traffic flow models. Netw. Spat. Econ..

[6] Gasser I. (2003). On non-entropy solutions of scalar conservation laws for traffic flow. J. Appl. Math. Mech..

[7] Machado J.A.T., Mata M.E. (2015). A fractional perspective to the bond graph modelling of world economies. Nonlinear Dyn..

[8] Carvalho A., Pinto C.M.A. (2017). A delay fractional order model for the co-infection of malaria and HIV/AIDS. Int. J. Dynam. Control.

[9] Zhou Y., Ionescu C., Machado T.J.A. (2015). Fractional dynamics and its applications. Nonlinear Dyn..

[10] Kumar D., Singh J., Baleanu D. (2018). A New Numerical Algorithm for Fractional Fitzhugh-Nagumo Equation Arising in Transmission of Nerve Impulses. Nonlinear Dyn..

[11] Kumar D., Singh J., Baleanu D. (2018). Analysis of regularized long-wave equation associated with a new fractional operator with Mittag-Leffler type kernel. Phys. A Stat. Mech. Appl..

[12] Singh J., Kumar D., Baleanu D. (2017). On the analysis of chemical kinetics system pertaining to a fractional derivative with Mittag-Leffler type kernel. Chaos Interdiscip. J. Nonlinear Sci..

[13] Hristov J. (2016). Transient heat diffusion with a non-Singular fading memory: From the Cattaneo constitutive equation with Jeffrey’s kernel to the Caputo-Fabrizio time-fractional derivative. Therm. Sci..

[14] Yang X.J., Machado J.A.T., Baleanu D. (2016). On exact traveling-wave solutions for local fractional Korteweg-de Vries equation. Chaos Interdiscip. J. Nonlinear Sci..

[15] Area I., Batarfi H., Losada J., Nieto J.J., Shammakh W., Torres A. (2015). On a fractional order Ebola epidemic model. Adv. Differ. Equ..

[16] Zaky M.A., Machado Z.A.T. (2017). On the formulation and numerical simulation of distributed-order fractional optimal control problems. Commun. Nonlinear Sci. Numer. Simul..

[17] Drapaca C.S., Sivaloganathan S. (2012). A fractional model of continuum mechanics. J. Elast..

[18] Sumelka W., Blaszczyk T., Liebold C. (2015). Fractional Euler–Bernoulli beams: Theory, numerical study and experimental validation. Eur. J. Mech. A Solid.

[19] Lazopoulos K.A., Lazopoulos A.K. (2017). Fractional vector calculus and fluid mechanics. J. Mech. Behav. Mater..

[20] Rahimi Z., Rezazadeh G., Sumelka W., Yang X.-J. (2017). A study of critical point instability of micro and nano beams under a distributed variable-pressure force in the framework of the inhomogeneous non-linear nonlocal theory. Arch. Mech..

[21] Yang X.J. (2012). Advanced Local Fractional Calculus and Its Applications.

[22] Yang X.J., Baleanu D., Srivastava H.M. (2015). Local fractional similarity solution for the diffusion equation on cantor set. Appl. Math. Lett..

[23] Yang X.J., Gao F., Srivastava H.M. (2017). Exact travelling wave solutions for the local fractional two-dimensional Burgers-type equations. Comput. Math. Appl..

[24] Yang X.J., Srivastava H.M., Torres D.F., Zhang Y. (2017). Non-differentiable Solutions for Local Fractional Nonlinear Riccati Differential Equations. Fundam. Inform..

[25] Yang X.J., Gao F. (2017). A New Technology for Solving Diffusion and Heat Equations. Therm. Sci..

[26] Yang X.J., Machado J.A.T., Haristov J. (2016). Nonlinear dynamics for local fractional Burgers’ equation arising in fractal flow. Nonlinear Dyn..

[27] Li Y., Wang L.F., Zeng S.D., Zhao Y. (2014). Local fractional Laplace variational iteration method for fractal vehicular traffic flow. Adv. Math. Phys..

[28] Zassim H.K. (2017). On approximate methods for fractal vehicular traffic flow. TWMS J. App. Eng. Math..

[29] Zhao D., Singh J., Kumar D., Rathore S., Yang X.J. (2017). An efficient computational technique for local fractional heat conduction equations in fractal media. J. Nonlinear Sci. Appl..

[30] Singh J., Kumar D., Nieto J.J. (2016). A reliable algorithm for local fractional Tricomi equation arising in fractal transonic flow. Entropy.

[31] Kumar D., Singh J., Baleanu D. (2017). A hybrid computational approach for Klein- Gordon equations on Cantor sets. Nonlinear Dyn..

[32] Jafari H., Jassim H.K., Moshokoa S.P., Ariyan V.M., Tchier F. (2016). Reduced differential transform method for partial differential equations within local fractional derivative operators. Adv. Mech. Eng..

[33] Keskin Y., Oturanc G. (2009). Reduced differential transform method for partial differential equations. Int. J. Nonlinear Sci. Numer. Simul..

[34] He J.H. (1999). Homotopy perturbation technique. Comput. Methods Appl. Mech. Eng..

[35] He J.H. (2003). Homotopy perturbation method: A new nonlinear analytical technique. Appl. Math. Comput..

[36] Yang X.J., Srivastava H.M., Cattani C. (2015). Local fractional homotopy perturbation method for solving fractal partial differential equations arising in mathematical physics. Rom. Rep. Phys..

[37] Srivastava H.M., Golmankhaneh A.K., Baleanu D., Yang X.J. (2014). Local fractional Sumudu transform with application to IVPs on Cantor sets. Abstr. Appl. Anal..

[38] Watugala G.K. (1993). Sumudu transform—A new integral transform to solve differential equations and control engineering problems. Int. J. Math. Edu. Sci. Technol..

[39] Belgacem F.B.M., Karaballi A.A., Kalla S.L. (2003). Analytical investigations of the Sumudu transform and applications to integral production equations. Math. Probl. Eng..

[40] Mittag-Leffler G.M. (1903). Sur la nouvelle fonction E_α_(x). Comptes Rendus Acad. Sci. Paris.

